# Tumor Lysis Syndrome: A Rare Complication of Chemotherapy for Metastatic Breast Cancer

**DOI:** 10.7759/cureus.4024

**Published:** 2019-02-07

**Authors:** Hafiz M Aslam, Cassandra Zhi, Sara L Wallach

**Affiliations:** 1 Internal Medicine, Seton Hall University, Hackensack Meridian School of Medicine, Trenton, USA; 2 Internal Medicine, Drexel University College of Medicine, Philadelphia, USA; 3 Internal Medicine, Seton Hall University / Hackensack Meridian School of Medicine, Trenton, USA

**Keywords:** solid tumors, tumor lysis syndrome, chemotherapy

## Abstract

Tumor lysis syndrome (TLS) is a fatal complication of chemotherapy treatment. It is rarely seen in the treatment of solid tumors particularly in breast cancer. We presented the case of a chemo-naïve 58-year-old Caucasian woman who developed tumor lysis syndrome (TLS) after a single treatment dose of gemcitabine for metastatic breast cancer. Despite optimal management, the patient clinically deteriorates and is referred to inpatient hospice. Although targeted chemotherapy options have become increasingly effective, physicians should be aware of the rare, yet often fatal complications of TLS. Similarly, physicians should be able to quickly recognize the development of TLS to ensure swift and effective prophylaxis or treatment.

## Introduction

Tumor lysis syndrome (TLS) is defined as an oncologic emergency, characterized by massive tumor cell lysis and the release of large amounts of potassium, phosphate, and nucleic acids into the systemic circulation. It is often seen as a result of chemotherapy treatment of lymphomas and T-cell acute lymphoblastic leukemias [1]. In one report, the incidence of TLS in AML was found to be around 17 percent. The incidence of TLS in solid tumors is very rare and is mostly described in case reports [1]. TLS is rarely observed in solid tumors such as breast cancer. In this report, we have described a rare case of TLS that occurred after a single treatment of gemcitabine, which only rarely causes TLS in solid tumors, in the context of metastatic breast cancer in a chemo-naïve patient [1] .

## Case presentation

A 58-year-old Caucasian woman was admitted to our hospital with complaints of generalized weakness, lethargy, anorexia, and weight loss. She was diagnosed with metastatic breast cancer 17 days prior to this admission. She also had a past medical history of treated hypertension and chronic back pain. Before this admission, she had complained of a breast lump in the previous year but never got it examined. The primary breast tumor was found on ultrasound to be approximately 4 cm by 5 cm and was found to be an invasive, poorly differentiated, ductal carcinoma with extensive necrosis. It had no expression of the hormone receptors, estrogen, and progesterone and was human epidermal growth factor 2 (HER2) positive. At the time of presentation, the cancer was advanced with innumerable hepatic metastases and multiple bilateral pulmonary metastases. There was also a small-moderate right pleural effusion. It had spread to the spine, causing a bony lytic lesion at the T9 vertebrae. On physical examination, she also had jaundice of the skin and mild splenomegaly, likely secondary to extensive liver disease.

The patient had undergone the planned chemotherapy four days prior, which was a treatment of gemcitabine 1600 mg. Gemcitabine has long been shown to be an effective agent in the treatment of metastatic breast cancer [2]. A Port-A-Cath had been placed successfully without any complications two days before the first chemotherapy treatment. On this present admission, her blood tests showed high uric acid levels (18.2 mg/dL), hyperphosphatemia (6.7 mg/dL), hyperkalemia (5.4 mmol/L), calcium (9.6 mg/dL), increased creatinine (3.38 mg/dL) and decreased glomerular filtration rate (14 mL/min). Nephrologists were consulted and they recognized this as TLS. It was recommended to give the patient vigorous intravenous (IV) fluid hydration with normal saline at 125 cc/hr as well as transfuse packed red blood cells to maintain the hemoglobin levels above 8 g/dL. Allopurinol 100 mg three times a day was also given. Hematologists/oncologists were consulted and they recommended chemotherapy treatment to be on hold for now until the patient’s labs become more stable.

By day two of admission, the patient appeared jaundiced and lethargic but was still alert and oriented. Her blood tests showed high uric acid levels (15.1 mg/dL), hyperphosphatemia (6.1 mg/dL), potassium (4.7 mmol/L), calcium (9.0 mg/dL), increased creatinine (2.69 mg/dL), and decreased glomerular filtration rate (18 mL/min). Rasburicase was not started at this time because it was not readily available at the current medical facility. Over the course of the next few days, the patient’s platelet count continued to drop, likely due to the initial chemotherapy treatment. The creatinine levels remained elevated, and the patient’s bilirubin and other liver function enzymes continued to rise, making the option of chemotherapy less feasible.

By day six of admission, the patient’s blood tests showed high uric acid levels (11.1 mg/dL), potassium (4.0 mmol/L), calcium (8.7 mg/dL), increased creatinine (2.71 mg/dL), and decreased glomerular filtration rate (18 mL/min). On examination, clinical deterioration was evident and the patient appeared even more lethargic and sleepy. She was difficult to wake with verbal stimuli.

Despite optimal management, by day seven of admission, she was drowsy and minimally responsive and had a slow response to any stimuli. At times, she could not open her eyes. At this time, it was decided by the patient, husband, and daughter that the patient would have a ‘do not resuscitate’ order and would be transferred to inpatient hospice when stable.

## Discussion

Only a few published cases of TLS developing in patients with breast cancer either due to underreporting or rarity are available. Moreover, the reason why TLS is rarely seen in solid tumors is currently unclear. TLS is more likely to happen in more indolent proliferations such as that seen in leukemias or lymphomas [3]. In a report evaluating the presence of TLS in breast cancer patients, it was found that in the few cases that have been published, most of the patients had metastatic breast adenocarcinomas. The age of the patients ranged from 31 to 94 years, with the average age being 54.1 years. The majority of these patients had a baseline increase in LDH as well as a baseline increase in uric acid levels [4].

TLS is diagnosed both clinically and through laboratory values. The Cairo-Bishop definition was proposed in 2004, which provides specific criteria for the diagnosis of TLS [5]. Clinically, the symptoms associated with TLS reflect the metabolic abnormalities. Symptoms include nausea, vomiting, diarrhea, anorexia, lethargy, hematuria, heart failure, cardiac dysrhythmias, seizures, muscle cramps, tetany, syncope, and possible sudden death [6]. Clinical TLS is defined as laboratory TLS plus one or more of the following: increased serum creatinine concentration (>1.5 times the upper limit of normal, cardiac arrhythmia/sudden death, or seizures). TLS can also be confirmed through laboratory values. Laboratory TLS is defined as two or more abnormal serum values, as shown in Table 1, presenting within three days before or seven days after chemotherapy treatment [5]. Based on these definitions, the patient, in this case, can be diagnosed with TLS both in terms of laboratory TLS and clinical TLS.

**Table 1:** Cairo-Bishop definition of laboratory tumor lysis syndrome NOTE: Two or more laboratory changes within three days before or seven days after cytotoxic therapy.

Element	Value	Change from baseline
Uric acid	≥476 micromol/L (8 mg/dL)	25% increase
Potassium	≥6.0 mmol/L (or 6 mEq/L)	25% increase
Phosphorus	≥2.1 mmol/L (6.5 mg/dL) for children or ≥1.45 mmol/L (4.5 mg/dL) for adults	25% increase
Calcium	< 1.75 mmol/L (7mg/ dL)	25% decrease

The prevalence of TLS occurring after chemotherapy with solid tumors is rare but may be underreported in many patients [7]. In recent years, there have been a few case reports about TLS developing in patients with breast cancer. For example, in 2012 and 2014, two studies were conducted that reviewed modern literature on TLS in solid tumors and included 100 and 120 patients with solid tumors complicated with TLS, respectively [1,8]. A literature search for metastatic breast cancer treated with gemcitabine, as in this case, complicated by TLS, showed only one or two cases prior to this present case [9]. This again indicated either the rarity of TLS occurring with the treatment of solid tumors or its underreporting and underrecognition.

Most patients who received chemotherapy treatment for solid tumors did not develop TLS. A strong risk factor for TLS is the patients’ health status. This includes the presence of hypotension, dehydration, acidic urine, oliguria, and nephropathy [10]. Certain medications may be additional risk factors for TLS due to their side effects of increasing uric acid levels in the body. These are shown in Table 2. The patient discussed in this case was not on any of these substances. Lastly, additional risk factors for TLS include the tumor’s size and expansion. For example, bulky tumors with wide metastatic dispersal and bone marrow involvement would put a patient at a higher risk [10]. In the present case, the patient had large primary breast cancer with numerous metastatic tumors to the liver, lungs, and spine, putting her at higher risk for the development of TLS.

**Table 2:** Medications/drugs that can increase the risk of tumor lysis syndrome by increasing uric acid levels in the body

Medications:		
Alcohol	Diazoxide	Methyldopa
Ascorbic acid	Diuretics (Thiazide)	Nicotinic acid
Aspirin	Epinephrine	Pyrazinamide
Caffeine	Ethambutol	Phenothiazines
Cisplatin	Levodopa	Theophylline

Prophylaxis with rasburicase or allopurinol is often initiated for patients with lymphomas or acute leukemias for prevention of TLS [6]. In a phase III trial comparing the use of rasburicase with allopurinol, 280 patients with hematologic malignancies at risk for TLS were assigned to be prophylaxed with rasburicase (0.2 mg/kg daily), rasburicase (0.2 mg/kg daily), and oral allopurinol (300 mg daily), or allopurinol alone (300 mg daily). The results showed that both rasburicase groups were superior to allopurinol alone in controlling serum uric acid levels. Rasburicase and allopurinol together were shown to contribute to a higher control of uric acid levels rather than rasburicase alone. However, this result was not statistically significant (*p *= 0.06) [11]. Rasburicase has been seen to be a more effective way of reducing hyperuricemia and has thus begun to replace allopurinol as prophylaxis. Because the incidence of TLS with solid tumors is less common, prophylaxis is often not done. Patients who develop TLS during chemotherapy should receive intensive supportive care with continuous cardiac monitoring and measurement of electrolytes and creatinine and uric acid levels every four to six hours. It is also important to treat specific electrolyte abnormalities, to administer rasburicase at 0.2 mg/kg, and to wash out obstructing uric acid crystals with fluids with or without a loop diuretic [1]. It should be noted that patients with solid tumors at a higher risk for TLS could also benefit from prophylaxis with rasburicase or allopurinol

## Conclusions

In this report, we describe one of the first literature-documented cases of TLS in a patient diagnosed with metastatic breast cancer as a result of a single dose of gemcitabine treatment. Although TLS is not an extremely common outcome resulting from chemotherapy treatment in patients with solid tumors, it is important for physicians to recognize patients who may be at a higher risk of developing TLS. Proper knowledge of the prevalence and outcomes is important for physicians to help recognize and prevent potentially fatal outcomes. Physicians should be aware of the clinical and laboratory diagnostic criteria of TLS as well as the options for management of TLS.
